# *In Vivo* Imaging of Tau Pathology Using Magnetic Resonance Imaging Textural Analysis

**DOI:** 10.3389/fnins.2017.00599

**Published:** 2017-11-06

**Authors:** Niall Colgan, Balaji Ganeshan, Ian F. Harrison, Ozama Ismail, Holly E. Holmes, Jack A. Wells, Nick M. Powell, James M. O'Callaghan, Michael J. O'Neill, Tracey K. Murray, Zeshan Ahmed, Emily C. Collins, Ross A. Johnson, Ashley Groves, Mark F. Lythgoe

**Affiliations:** ^1^Division of Medicine, UCL Centre for Advanced Biomedical Imaging, University College London, London, United Kingdom; ^2^School of Physics, National University of Ireland Galway, Galway, Ireland; ^3^Institute of Nuclear Medicine, University College London Hospitals, London, United Kingdom; ^4^Eli Lilly & Co. Ltd., Windlesham, United Kingdom; ^5^Eli Lilly & Co. Ltd., Lilly Corporate Center, Indianapolis, IN, United States

**Keywords:** texture analysis, Alzheimer's Disease, MRTA, MRI imaging, tauopathies

## Abstract

**Background:** Non-invasive characterization of the pathological features of Alzheimer's disease (AD) could enhance patient management and the development of therapeutic strategies. Magnetic resonance imaging texture analysis (MRTA) has been used previously to extract texture descriptors from structural clinical scans in AD to determine cerebral tissue heterogeneity. In this study, we examined the potential of MRTA to specifically identify tau pathology in an AD mouse model and compared the MRTA metrics to histological measures of tau burden.

**Methods:** MRTA was applied to T2 weighted high-resolution MR images of nine 8.5-month-old rTg4510 tau pathology (TG) mice and 16 litter matched wild-type (WT) mice. MRTA comprised of the filtration-histogram technique, where the filtration step extracted and enhanced features of different sizes (fine, medium, and coarse texture scales), followed by quantification of texture using histogram analysis (mean gray level intensity, mean intensity, entropy, uniformity, skewness, standard-deviation, and kurtosis). MRTA was applied to manually segmented regions of interest (ROI) drawn within the cortex, hippocampus, and thalamus regions and the level of tau burden was assessed in equivalent regions using histology.

**Results:** Texture parameters were markedly different between WT and TG in the cortex (E, *p* < 0.01, K, *p* < 0.01), the hippocampus (K, *p* < 0.05) and in the thalamus (K, *p* < 0.01). In addition, we observed significant correlations between histological measurements of tau burden and kurtosis in the cortex, hippocampus and thalamus.

**Conclusions:** MRTA successfully differentiated WT and TG in brain regions with varying degrees of tau pathology (cortex, hippocampus, and thalamus) based on T2 weighted MR images. Furthermore, the kurtosis measurement correlated with histological measures of tau burden. This initial study indicates that MRTA may have a role in the early diagnosis of AD and the assessment of tau pathology using routinely acquired structural MR images.

## Introduction

The pathophysiological processes in Alzheimer's disease (AD), and the formation of the two pathogenic hallmarks of the disease (amyloid β and tau accumulations Morris et al., 2001), are thought to begin many years before clinical diagnosis (Jack et al., 2010). Accumulation of such proteinopathies is associated with neurodegeneration and cognitive decline in AD (Braak and Braak, 1991). Therefore non-invasive detection of the pathological features of AD in neural tissue could provide early detection of the anatomical regions affected, and aid in the management of patients and development of novel therapies (Jack et al., 2013). Structural magnetic resonance imaging (MRI) has become an integral part of the clinical assessment of patients where reductions in volume and shape changes of the medial temporal structures are now considered to be a diagnostic marker of the early, mild cognitive impairment stage of AD development (Frisoni et al., 2010). Additionally, quantitative magnetic resonance imaging texture analysis (MRTA) has been used to derive heterogeneity information in cerebral tissue by extracting texture descriptors from structural MR in AD (Freeborough and Fox, 1998; Torabi et al., 2006; De Oliveira et al., 2011) as well as other neurological disorders (Ganeshan et al., 2010; Radulescu et al., 2013a; Sanz-Cortes et al., 2013; Suoranta et al., 2013). It is currently unknown, however precisely how these texture descriptors relate to tau pathology, one of the two major proteinopathies associated with AD development.

In this study we applied MRTA to T2 weighted structural MRI scans of an animal model of tau pathology, the rTg4510 (TG) mouse model of tauopathy (Ramsden et al., 2005) and litter matched wild-type (WT) mice at 8.5 months. The TG mouse model over expresses a mutant form of human tau (P301L) resulting in tau accumulation in the form of neurofibrillary tangles (NFTs) largely restricted to the hippocampus, cortex, olfactory bulb, and striatum (Santacruz et al., 2005). Many approaches have been developed for extracting texture characteristics from digital images, such as transform-based, structural-based, and statistical-based methods (Meyer-Baese and Schmid, 2014). In this study, we used the combination of transform- and statistical-based image filtration-histogram approaches to extract texture features (Miles et al., 2012). This filtration-histogram approach, which previously demonstrated diagnostic-potential in schizophrenia (Ganeshan et al., 2010; Radulescu et al., 2014) and Asperger syndrome (Radulescu et al., 2013a,b), uses a filtration-step which amplifies/enhances features (or objects) of different sizes (at the fine, medium, coarse texture scale) followed by quantification using histogram analysis to detect changes in pixel gray-level distribution or heterogeneity (e.g., intensity, irregularity, skewness, and sharpness of histogram). As such the changes in the heterogeneity of tissue detected by MRTA could reflect the degree of AD pathology in neural tissue (De Oliveira et al., 2011).

The aims of this study are (i) to determine if MRTA can detect tau pathology in the rTg4510 mouse and (ii) compare MRTA metrics with histological measures of tau burden across three brain regions with different tau loading.

## Materials and methods

### Animals

Generation of homozygous rTg4510 transgenic mice has been reported previously (Ramsden et al., 2005). Mice were licensed from the Mayo Clinic (Jacksonville Florida, USA) and bred for Eli Lilly by Taconic (Germantown, USA). Mice were imported to the UK for imaging studies at the Centre for Advanced Biomedical Imaging (CABI), London. All studies were carried out in accordance with the United Kingdom Animals (Scientific Procedures) Act of 1986. Sixteen wild-types (WT) and nine transgenic strain rTg4510 (TG) 8.5-month-old female litter matched mice were imaged *in vivo*. After completion of imaging studies, animals were perfuse fixed and brains removed for histology.

### MRI

*In vivo* experiments were performed on a 9.4T Agilent horizontal bore scanner and VnmrJ version 3.1a front end software. The animals were placed in an induction chamber and anesthetized with inhaled isoflurane (2% isoflurane at 1 L/mO_2_) until pedal withdrawal reflex was lost. They were then transferred to an MRI cradle to minimize motion artifacts and maintained at 1.5% isoflurane at 1 L/mO_2_ for the duration of scanning. Core temperature was maintained at 37° Celsius using a warm air blower feedback system and rectal probe (SA instruments). Physiological monitoring of temperature and respiration was recorded throughout the scan (SA instruments). RF transmission was performed with a 72 mm volume coil and a 4 channel receiver coil (Rapid biomedical). A T2 weighted, 3D fast spin-echo sequence was implemented with FOV = 19.2 × 16.8 × 12.0 mm; resolution = 150 × 150 × 150 μm; TR = 2,500 ms, TEeff = 43 ms, ETL = 4; NSA = 1. Once scanning was completed, animals were perfuse fixed and the brain of each TG and WT mouse was removed for histology.

### Texture analysis

**Figure 1:**
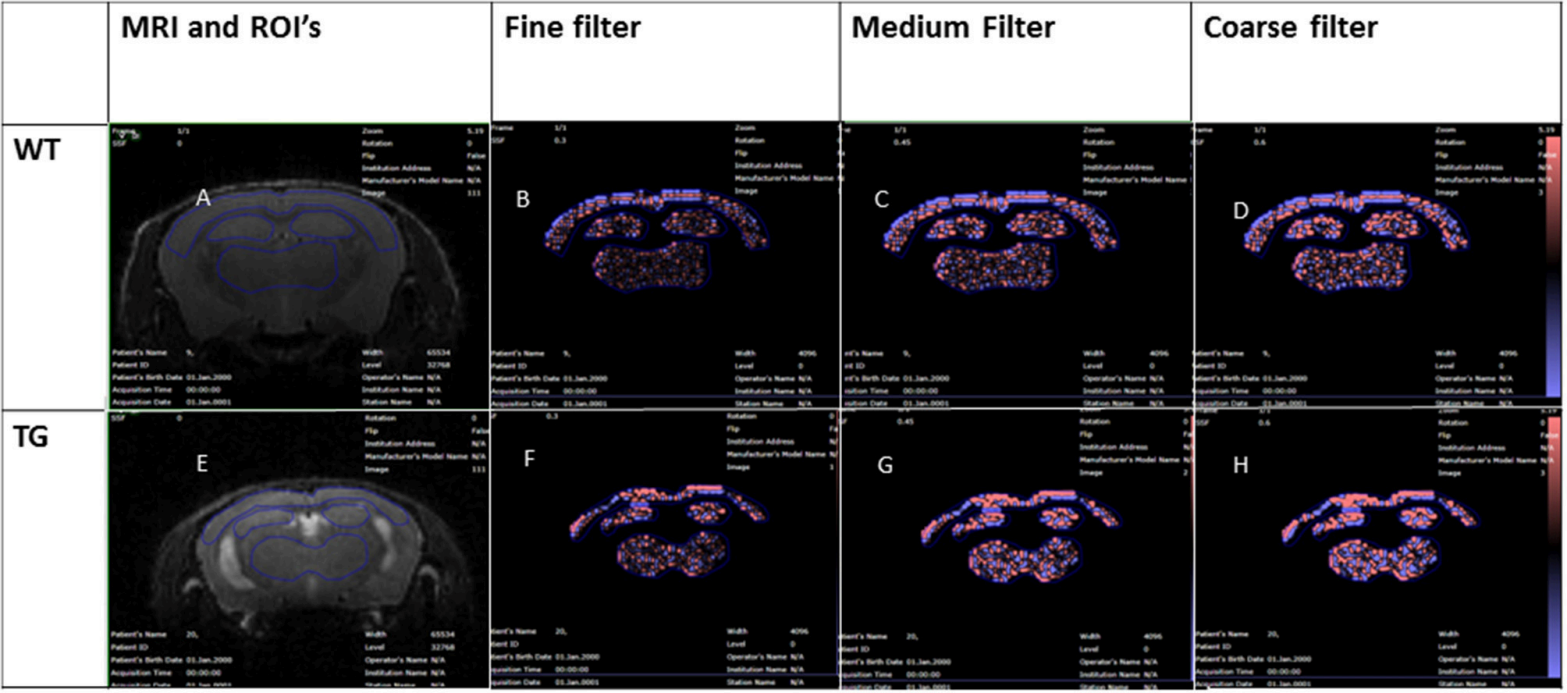
Wildtype and rTg4510 texture analysis images, MRI image with ROI's **(A,E)**, fine filter images **(B,F)**, medium filter **(C,G)**, and coarse filter **(D,H)**.

Each volume scan was corrected for intensity non-uniformity using the N3 method (Sled et al., 1998) and imported into TexRAD research software (TexRAD Ltd, part of Feedback Plc, Cambridge, UK) to undertake MRTA. For each mouse, two-dimensional regions of interest (ROI) were manually drawn in the cortex, hippocampus and thalamus (Figures 1A,E) on images in the slice location that aligned with the anatomical location of histology (−2 mm from bregma). The three ROIs were manually drawn by a senior imaging scientist (with more than 9 years of experience in texture-analysis) who was blinded for genotype.

The inter-pixel variability within each region of interest is assessed by filtering each region into fine, medium and coarse features denoted by the Spatial Scaling Factor (SSF), which for our study, detects structures in radii of 0.3, 0.6, and 0.9 mm respectively (14). The histograms of the pixel values in the filtered images are then quantified using standard descriptors, specifically: mean gray level intensity (mean intensity) (M), entropy (E), uniformity (U), skewness (S), and kurtosis (K). MRTA consisted of a filtration-histogram technique (14, 15) where the image filtration was performed using a Laplacian of Gaussian (LoG) band-pass filter producing a series of derived images highlighting features at different anatomical spatial scales ranging from fine, medium and coarse texture for the ROI (Ganeshan et al., 2007). The different spatial scale filter (SSF) values used in this study were fine texture (corresponding to features of 0.3 mm in radius), medium textures (corresponding to features of 0.6 mm in radius) and coarse texture (corresponding to features of 0.9 mm in radius) scales as highlighted in both WT (Figures 1B–D) and TG (Figures 1F–H) respectively. This was followed by quantification of each of these filtered images using histogram analysis of which comprised measuring six parameters: mean gray level intensity (mean intensity) (M), entropy (E), uniformity (U), skewness (S), standard-deviation (*SD*), and kurtosis (K) (Miles et al., 2012). A detailed explanation of what texture parameters are and how they relate to the components of image heterogeneity has been previously reported (Miles et al., 2012). However, in brief, the mean gray level intensity reflects the average value of the pixels within the region of interest. Entropy measures the irregularity/disorder of interpixel intensities at each filter size. Low uniformity indicates an increase in tissue heterogeneity. Skewness determines the symmetry of profile of the image intensity. The standard deviation measures how much variation or “dispersion” exists from the average. Kurtosis is a measure of the “pointiness” or “sharpness” of histogram distribution which has previously been demonstrated to detect variability in tissue hyper intensities (Miles et al., 2012).

### Histology

Brain samples were processed using the Tissue TEK® VIP processor (GMI Inc., MN, USA). Sections were embedded in paraffin wax to allow coronal brain sections to be cut. Serial sections (6–8 μm) were taken using HM 200 and HM 355 (Thermo Scientific Microm, Germany) rotary microtomes. Immunohistochemistry (IHC) was performed using a primary antibody for tau phosphorylated at serine 409 (PG-5; 1:500 from Peter Davies, Albert Einstein College of Medicine, NY, USA). The secondary antibody was applied and slides were then incubated with avidin biotin complex (ABC) reagent for 5 min (M.O.M. kit PK-2200, Elite ABC rabbit kit PK-6101, or Elite RTU ABC PK-7100 Vector Labs). After rinsing, slides were treated with the chromogen 3,3′-diaminobenzidine (DAB; Vector Laboratories, SK-4100) to allow visualization. The slides were then cover slipped, dried and digitized using an Aperio Scanscope XT (Aperio Technologies Inc., CA, USA).

Images were viewed and analyzed with Aperio ImageScope software (version 10.2.2319). In this study, sections that align with the MRI images for each mouse (−2 mm from bregma) were analyzed. For each section stained, areas of specific interest (in this case the cortex, hippocampus, and thalamus) were delineated. The density of PG-5 immunoreactivity (immunoreactive cells/mm^2^) was quantified using Aperio ImageScope and exported into SPSS 19.0 (IBM SPSS, Chicago, Illinois) for statistical comparison with MRTA measurements.

### Statistical analysis

Independent *T*-tests were used to determine group differences for each texture parameter between WT and TG. To correct for multiple testing, a false discovery rate (FDR) correction with a significance level of 0.05 was applied. Prior to correlating with histological measures of tau burden, an exclusion criterion was applied to the data, where texture parameters that did not significantly differ between the two groups across all three anatomical regions at the same SSF value were excluded from the further evaluation of its correlation with histology using Pearson's correlation. Pearson correlations of texture parameters with PG-5 immunoreactivity to tau pathology were also carried out. All statistical analyses were performed using SPSS 19.0 (IBM SPSS, Chicago, Illinois) and a *p*-value of 0.05 and below was considered to be significant.

## Results

### Histology

**Figure 2:**
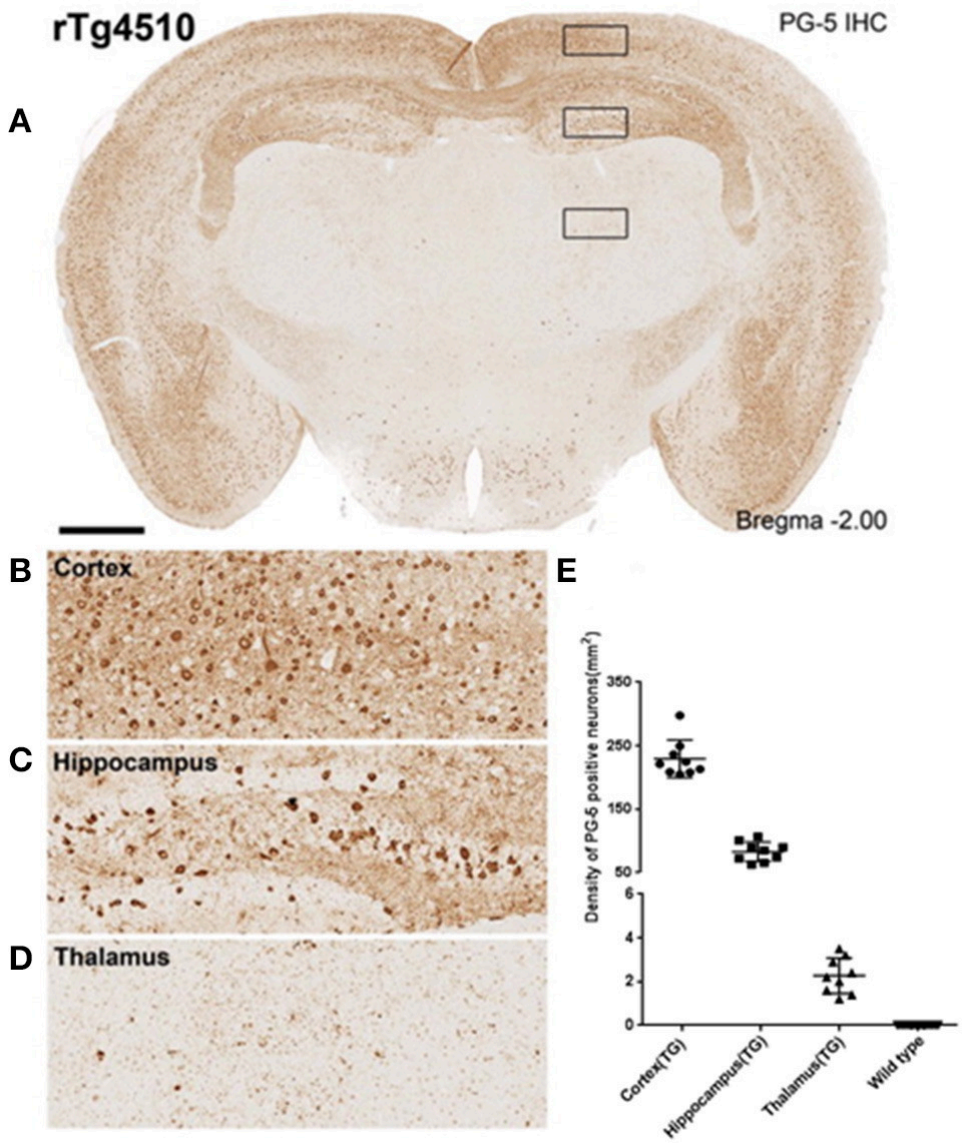
Immunohistochemistry to estimate regional PG-5 positive NFT density. **(A)** Single slice from a representative rTg4510 mouse with staining for PG-5 positive NFTs. Marked regional dependence of NFT density is observable [see inset **(B–D)** for examples of cortical **(B)**, hippocampal **(C)**, and thalamic **(D)** NFT distribution]. **(E)** Quantitative regional estimates of NFT density for each of the 16 WT and 9 TG mice that underwent MRI (8.5 month age).

In TG animals, each anatomical region selected for analysis had a varying degree of tau pathology (Figures 2A–D). The cortex, with an average cell density count of 229 ± 10 stained for filamentous tau, being the most heavily affected, then the hippocampus at 83 ± 5 and lastly the thalamus at 2.3 ±0.3 (Figure 2E). As expected, WT animals did not display tau pathology.

### MRTA

#### Cortex

In the cortex (a region with the highest tau burden, Figure 2E) several MRTA metrics significantly differentiated the TG animals from the WT group. The TG group had significantly lower entropy whereas kurtosis, mean intensity, uniformity, and skewness were significantly higher in TG group. In the cortex, entropy, and kurtosis were significant across all three spatial scaling factors (SSF) of fine; medium and coarse (Table 1).

**Table 1:** Significant cortex textural parameters of mean gray level intensity (M), entropy (E), uniformity (U), skewness (S), and kurtosis (K).

**Parameter**	**Mean values (±SE)**		***P***	***t*-ratio**	***df***
	**rTg4510**	**Wildtype**			
M fine	4, 536 ± 0.49	4068 ± 0.33	0.0001	4.31	23.0
E fine	6.14 ± 0.01	6.57 ± 0.01	0.001	5.39	23.0
K fine	0.23 ± 0.06	−0.74 ± 0.11	0.001	4.04	23.0
E medium	4.54 ± 0.2	5.54 ± 0.07	0.001	4.59	23.0
U medium	0.05 ± 0.01	0.03 ± 0.01	0.04	4.76	23.0
K medium	0.26 ± 0.3	−0.69 ± 0.1	0.02	5.97	23.0
E coarse	0.66 ± 0.29	2.67 ± 0.28	0.001	5.69	23.0
U coarse	0.73 ± 0.12	0.22 ± 0.07	0.001	4.01	23.0
S coarse	0.48 ± 0.31	−0.61 ± 0.15	0.002	4.34	23.0
K coarse	0.11 ± 0.01	−0.42 ± 0.02	0.0001	6.34	23.0

The mean intensity (M, *p* = 0.0001) was significant at a fine feature level, uniformity was significant at both medium uniformity (*p* = 0.04) and coarse filter uniformity (*p* = 0.001) and skewness (*p* = 0.002) of the intensity profile were significant (Table 1).

#### Hippocampus

In the hippocampus (a region with the second highest tau burden, Figure 2E) there was a reduction in the number of significant MRTA metrics which differentiated the TG animals from the WT group (Table 2).

**Table 2:** Significant hippocampus textural parameters of entropy (E) and kurtosis (K).

**Parameter**	**Mean values (±SE)**		***P***	***t*-ratio**	***df***
	**rTg4510**	**Wildtype**			
E fine	5.34 ± 0.10	6.24 ± 0.09	0.001	9.34	23.0
K fine	0.59 ± 0.24	−0.22 ± 0.28	0.05	5.52	23.0
E medium	3.57 ± 0.30	4.72 ± 0.1	0.002	6.39	23.0
K medium	0.48 ± 0.07	−0.2 ± 0.07	0.001	4.36	23.0
K coarse	0.18 ± 0.02	−0.41 ± 0.01	0.001	4.34	23.0

However, there was a similar increase in kurtosis in TG compared to WT across all the three SSF values of fine (*p* = 0.05), medium (*p* = 0.001), and coarse (*p* = 0.001) texture features, and a decrease in entropy in TG compared to WT in only fine (*p* = 0.001) and medium (*p* = 0.002; Table 2) texture features.

#### Thalamus

In the thalamus (a region with the lowest tau burden, Figure 2E), there was also a reduction in the number of MRTA metrics that significantly differentiated TG from WT animals in comparison to the cortex region. Again kurtosis was significantly increased in the TG compared to WT animals across all three SSF values of fine (*p* = 0.001), medium (*p* = 0.004) and coarse (*p* = 0.001) texture features. Additionally, a decrease in entropy at the fine (*p* = 0.001) and medium (*p* = 0.002) texture features were also observed, whereas coarse uniformity (*p* = 0.009) was significantly higher in TG compared to WT (Table 3).

**Table 3:** Significant thalamus textural parameters of entropy (E), kurtosis (K), and uniformity (U).

**Parameter**	**Mean values (±SE)**		***P***	***t*-ratio**	***df***
	**rTg4510**	**Wildtype**			
E fine	6.28 ± 0.03	7.03 ± 0.02	0.001	9.35	23.0
K fine	0.09 ± 0.001	−0.1 ± 0.01	0.001	4.19	23.0
E medium	5.49 ± 0.004	6.19 ± 0.001	0.002	6.39	23.0
K medium	0.02 ± 0.002	−0.02 ± 0.003	0.004	4.36	23.0
K coarse	0.34 ± 0.007	−0.19 ± 0.002	0.001	4.33	23.0
U coarse	0.03 ± 0.001	0.02 ± 0.001	0.001	4.86	23.0

### Correlations between MRTA and tau burden

**Figure 3:**
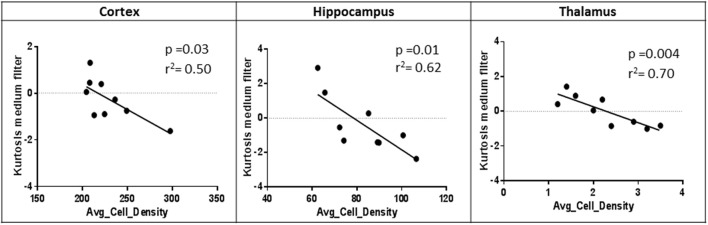
Pearson correlation of average cell density and Kurtosis (medium texture filter) in each anatomical region of rTg4510 mice (*n* = 9).

Correlation analyses were only performed on MRTA metrics that consistently differentiated TG from WT mice across all three anatomical regions (cortex, hippocampus, and thalamus). Entropy and Kurtosis were the MRTA metrics that consistently differentiated TG from WT mice across all three anatomical regions. However, only kurtosis at the medium texture scale demonstrated a clear association with degree of tau burden at the three anatomical regions (cortex, *p* = 0.02, hippocampus, *p* = 0.001, thalamus, *p* = 0.004; Figure 3). All textural measurements are provided in Supplementary Table 1.

## Discussion

In this study, we firstly applied MRTA in the rTg4510 mouse model to investigate its ability to non-invasively detect tau pathology in this mouse model in comparison to litter matched wild type control animals. Secondly, we sought to determine if any MRTA metrics have a specific correlation with the tau burden. We observed that MRTA highlighted cortical and sub-cortical textural abnormalities in regions of both high and low tau loading indicating this may be a sensitive marker of tauopathy.

Of the six textures, we found higher kurtosis in TG animals, which reflects a sharper histogram profile, indicating an accumulation of elevated intensities closely distributed near the mean intensity of the highlighted features of each SSF. This may reflect the subtle, differentially localized features of pathology associated with gray matter thinning in TG animals (Ludvigson et al., 2011), increases in large empty spherical vacuoles (Ludvigson et al., 2011) resulting in increased image intensities of the T2 weighted images of the TG animals, due to the increased NFT burden (Santacruz et al., 2005) at 8.5 months.

In the TG animals, we also noted that entropy was reduced in the cortex, hippocampus, and thalamus, a measure of the irregularity or complexity of the signal intensity (Tables 1–3). This reduction in tissue complexity was also seen as an increased uniformity in the cortex and thalamus, which could reflect an overall increase in the homogeneity of the tissue. Reductions of up to 80% in volume of the cortex, blurring of cortical layers (Ludvigson et al., 2011) and reduced dispersion in the cortex and hippocampus (Colgan et al., 2016), have been previously reported in the rTG4510 at 9 months old. A similar pathological effect in our study cohort may account for the reduced entropy in all three anatomical regions and increased uniformity in the cortex and thalamus of the TG group observed here.

The metrics that consistently differentiated the TG from WT groups in all three anatomical regions with varying degrees of tau pathology were kurtosis and entropy. To determine which of these metrics most closely related to the level of tau burden we correlated kurtosis and entropy with the histological measures of tau pathology in the TG mouse brain in each anatomical region. Kurtosis was the only metric that demonstrated a notable relationship with the histological measures of the degree of tau burden for each anatomical region across all the three filters—fine, medium, and coarse texture features. It has been previously reported that a higher degree of NFT burden is linked with increased neural tissue hyperintensities in T2 weighted MRI (Erten-Lyons et al., 2013) as a result of the empty vacuoles, up to 60 μm in diameter (Ludvigson et al., 2011). Extensive hyperintensities (from increased tissue vacuolation) would result in an increase in sharpness of histogram distribution within each SSF reflecting an increased kurtosis in the TG animals. This is particularly apparent in the cortex and hippocampus (Tables 1–3), the regions with the highest tau burden. The pathological processes leading to these hyperintensities occur in conjunction with tauopathology, and may explain the close relationship between NFT density and kurtosis.

Our findings suggest that MRTA metrics are associated with the level of tau burden in the mouse brain. In each anatomical region affected by varying degrees of tau burden, MRTA metrics differentiated between the TG and WT groups. Furthermore, there was a significant association between the extent of tau pathology and kurtosis in the TG tau animals. The ability to retrospectively characterize tau burden from initial and follow-up structural MRI scans would be an invaluable tool in the staging and monitoring of tauopathies, and the efficacy of therapeutic strategies. As such, the main strength of MRTA is that it can be easily applied to structural MR images, acquired routinely as part of preclinical MRI studies, and in clinical assessment. Future work will aim to validate our findings at earlier time points in a longitudinal study and to further develop the technique to examine 3D volumes of interest.

## Ethics statement

All studies were carried out in accordance with the UK Animals (Scientific Procedures) Act of 1986 and subject to approval by the internal ethical review panel of University College London. Animals were placed in an induction chamber and anesthetized with inhaled isoflurane (2% isoflurane at 1 L/mO_2_) until pedal withdrawal reflex was lost and maintained at 1.5% isoflurane at 1 L/mO_2_ for the duration of scanning. Physiological monitoring of temperature and respiration was recorded throughout the scan. Once scanning was completed, animals were perfuse fixed and the brain of each TG and WT mouse was removed for histology.

## Author contributions

NC and BG: Wrote the main manuscript text and performed the data analysis on the *in vivo* quantitative MRI data and quantitative histological data. NC, OI, and HH: Planned and carried out the study and performed all the *in vivo* experiments and data collection. BG: Performed the texture MRI image processing and analysis. IFH, JO, and JW: Provided intellectual input toward the analysis and interpretation of the *in vivo* MRI data. NP: Provided intellectual input toward the analysis and interpretation of MRI data. TM and ZA: Performed all histological experiments and data collection. MO, RJ, and EC: Provided all contributing authors with overall direction in this project. AG and ML are acknowledged as senior authors due to their significant intellectual input and direction.

### Conflict of interest statement

BG is a director, part-time employee and shareholder of Feedback Plc (Cambridge, England, UK), company that develops and markets the TexRAD texture analysis algorithm described in this manuscript. This work was produced in collaboration with Eli Lilly and Company and is part funded by Eli Lilly. The authors declare that the research was conducted in the absence of any commercial or financial relationships that could be construed as a potential conflict of interest.
