# Supplementary material for: In Vivo Imaging of Tau Pathology Using Magnetic Resonance Imaging Textural Analysis
Source: Front Neurosci. 2017 Nov 6;11:599. doi: 10.3389/fnins.2017.00599 (PMC5681716; doi:10.3389/fnins.2017.00599)
Supplement: Supplementary file 1 [file Table1.DOCX]

Supplementary Material

In Vivo Imaging of Tau Pathology Using Magnetic Resonance Imaging Textural Analysis

**N Colgan^1,5*α^, B Ganeshan^2 α^, IF Harrison^1^, O Ismail^1^, HE Holmes^1^, JA Wells^1^, N Powell^1^, JM O’Callaghan^1^, M O’Neill^3^, T Murray^3^, Z Ahmed^3^, EC Collins^4^, RA Johnson^4^, A Groves^2 ρ^, MF Lythgoe^1 ρ^**

*** Correspondence:** Corresponding Author: [niall.colgan@nuigalway.ie](mailto:niall.colgan@nuigalway.ie)

|  | Mean values (± SE) | | | |  |  |  |  |  |
| --- | --- | --- | --- | --- | --- | --- | --- | --- | --- |
| Parameter | rTg4510 | | Wild type | | P value | T ratio | df | Region |  |
| U fine | 0.0066 | ± 0.001 | 0.00067 | ±0.001 | P > 0.05 | 5.572e-005 | 23 | Cortex |  |
| S fine | 17.30 | ± 6.02 | 19.031 | ±2.64 | P > 0.05 | 0.01626 | 23 |  |  |
| SD fine | 3866.96 | ± 915.14 | 3053.12 | ±324.97 | P > 0.05 | 1.749 | 23 |  |  |
| M medium | 4336.33 | ± 1055.47 | 4068.4 | ±329.71 | P > 0.05 | 1.578 | 23 |  |  |
| S medium | 13.45 | ± 4.07 | 8.33 | ±2.06 | P > 0.05 | 0.04811 | 23 |  |  |
| SD medium | 1367.79 | ± 525.87 | 1579.85 | ±194.15 | P > 0.05 | 1.993 | 23 |  |  |
| M coarse | 4256.79 | ± 680.11 | 4666.44 | ±329.23 | P > 0.05 | 1.030 | 23 |  |  |
| SD coarse | 1079.60 | ± 243.59 | 982.57 | ±217.45 | P > 0.05 | 0.9117 | 23 |  |  |
| M fine | 111.81 | ±770.26 | 679.73 | ±47.54 | P > 0.05 | 0.6382 | 23 | Hippocampus |  |
| U fine | 0.012 | ±0.0015 | 0.01 | ±0.0001 | P > 0.05 | 1.879e-005 | 23 |  |  |
| S fine | 18.64 | ±4.93 | 36.25 | ±3.06 | P > 0.05 | 0.1655 | 23 |  |  |
| SD fine | 2443.43 | ±453.38 | 1398.95 | ±149.32 | P > 0.05 | 0.4179 | 23 |  |  |
| M medium | 2905.83 | ±727.99 | 1062.77 | ±125.56 | P > 0.05 | 0.4046 | 23 |  |  |
| U medium | 0.023 | ±0.0017 | 0.014 | ±0.0014 | P > 0.05 | 8.456e-005 | 23 |  |  |
| S medium | 7.84 | ±3.01 | 9.79 | ±1.89 | P > 0.05 | 0.01832 | 23 |  |  |
| SD medium | 1819.90 | ±893.68 | 959.89 | ±137.46 | P > 0.05 | 1.315 | 23 |  |  |
| M coarse | 2909.57 | ±578.28 | 1944.21 | ±181.75 | P > 0.05 | 1.554 | 23 |  |  |
| E coarse | 4.55 | ±0.24 | 5.30 | ±0.0546 | P > 0.05 | 0.007047 | 23 |  |  |
| U coarse | 0.05 | ±0.008 | 0.03 | ±0.0013 | P > 0.05 | 0.0001879 | 23 |  |  |
| S coarse | 7.95 | ±3.67 | 9.37 | ±2.39 | P > 0.05 | 0.01334 | 23 |  |  |
| SD coarse | 1676.30 | ±694.19 | 835.53 | ±99.22 | P > 0.05 | 2.262 | 23 |  |  |
| M fine | 123.20 | ±97.11 | 221.79 | ±22.3975 | P > 0.05 | 0.9263 | 23 | Thalamus |  |
| U fine | 0.0067 | ±0.0018 | 0.000667 | ±0.00067 | P > 0.05 | 5.669e-005 | 23 |  |  |
| S fine | 44.15 | ±2.36 | 45.16 | ±1.26 | P > 0.05 | 0.009490 | 23 |  |  |
| SD fine | 1232.76 | ±132.36 | 1090.24 | ±72.69 | P > 0.05 | 1.339 | 23 |  |  |
| M medium | -198.23 | ±146.29 | -50.80 | ±47.65 | P > 0.05 | 1.385 | 23 |  |  |
| U medium | 0.012 | ±0.0016 | 0.0093 | ±0.000667 | P > 0.05 | 2.537e-005 | 23 |  |  |
| S medium | 29.82 | ±3.60 | 31.10 | ±1.98 | P > 0.05 | 0.01203 | 23 |  |  |
| SD medium | 1137.22 | ±216.62 | 1073.16 | ±74.59 | P > 0.05 | 0.6019 | 23 |  |  |
| M coarse | -573.04 | ±188.07 | -500.35 | ±54.81 | P > 0.05 | 0.6830 | 23 |  |  |
| E coarse | 5.8 | ±0.09 | 6.32 | ±0.12 | P > 0.05 | 0.004886 | 23 |  |  |
| S coarse | 23.38 | ±2.99 | 24.93 | ±1.86 | P > 0.05 | 0.01456 | 23 |  |  |
| SD coarse | 1092.13 | ±232.00 | 1017.82 | ±69.24 | P > 0.05 | 0.6982 | 23 |  |  |

**Supplementary Table 1.**. Non-significant textural parameters in the cortex, hippocampus and thalamus.
